# Microstructural characterization of annulus fibrosus by ultrasonography: a feasibility study with an in vivo and in vitro approach

**DOI:** 10.1007/s10237-019-01189-3

**Published:** 2019-06-20

**Authors:** Tristan Langlais, Pierre Desprairies, Raphael Pietton, Pierre-Yves Rohan, Jean Dubousset, Judith R. Meakin, Peter C. Winlove, Raphael Vialle, Wafa Skalli, Claudio Vergari

**Affiliations:** 1 Arts et Métiers ParisTech, LBM/Institut de Biomécanique Humaine Georges Charpak, 151 bd de l’Hôpital, 75013 Paris, France; 2 Department of Pediatric Orthopaedics, Armand Trousseau Hospital, Sorbonne University, UPMC Paris 6 University, Paris, France; 3 School of Physics and Astronomy, University of Exeter, Exeter, UK

**Keywords:** Intervertebral disc, Lamellae, MRI, Microscopy, Geometry

## Abstract

The main function of the intervertebral disc is biomechanical function, since it must resist repetitive high loadings, while giving the spine its flexibility and protecting the spinal cord from over-straining. It partially owes its mechanical characteristics to the lamellar architecture of its outer layer, the annulus fibrosus. Today, no non-invasive means exist to characterize annulus lamellar structure in vivo. The aim of this work was to test the feasibility of imaging annulus fibrosus microstructure in vivo with ultrasonography. Twenty-nine healthy adolescents were included. Ultrasonographies of L3–L4 disc were acquired with a frontal approach. Annulus fibrosus was segmented in the images to measure the thickness of the lamellae. To validate lamellar appearance in ultrasonographies, multimodality images of two cow tail discs were compared: ultrasonography, magnetic resonance and optical microscopy. In vivo average lamellar thickness was 229.7 ± 91.5 μm, and it correlated with patient body mass index and age. Lamellar appearance in the three imaging modalities in vitro was consistent. Lamellar measurement uncertainty was 7%, with good agreement between two operators. Feasibility of ultrasonography for the analysis of lumbar annulus fibrosus structure was confirmed. Further work should aim at validating measurement reliability, and to assess the relevance of the method to characterize annulus alterations, for instance in disc degeneration or scoliosis.

## Introduction

Intervertebral disc is the largest avascular organ of the human body. Its main function is biomechanical function: it gives spine its flexibility while protecting the spinal cord from over-straining. Thus, it must undergo large and repetitive loads and absorb mechanical shocks. Disc compliance and resistance are strongly related to its complex structure: it is composed of a strong fibrous outer ring (*annulus fibrosus*, AF) retaining a gel-like substance in the middle (*nucleus pulposus*). The AF in particular is made of concentric fibrous lamellae, which are rich in collagen fibres running parallel within a given lamella. Each lamella runs in a different direction, and they tend to be discontinuous, i.e. they are not rings running around the whole disc, but rather bands connecting adjacent endplates (Marchand and Ahmed 1990; Vergari et al. 2017).

Given its importance in spinal biomechanics, the intervertebral disc is often a key element of numerical models of the spine and trunk (Dreischarf et al. 2014). Thus, the realism and the relevance of these models rely on the experimental characterization of disc’s mechanical and structural properties (Barthelemy et al. 2016). In particular, the attention has been shifting from generic modelling to patient-specific modelling and, thanks to the continuous improvement of calculation power, towards detailed multiscale modelling (Little and Adam 2015; Mengoni et al. 2015; Toumanidou and Noailly 2015; Kassab et al. 2016).

Disc mechanical properties can be estimated in vitro from mechanical testing or imaging-based methods, such as shearwave elastography based on ultrasound or magnetic resonance imaging (Vergari et al. 2014a; Ben-Abraham et al. 2017). In vivo, disc properties can be estimated with bending, suspension or fulcrum tests under radiological imaging (Hirsch et al. 2015; He and Wong 2018), or non-invasively through shearwave elastography (Streitberger et al. 2015; Vergari et al. 2016). However, characterizing disc microstructure with non-destructive methods is still a challenge. In vitro, high-resolution magnetic resonance imaging (MRI) and micro-computed tomography gives access to disc size and to the annulus’ lamellar structure (Lin and Tang 2017), but, to our knowledge, no means currently exists to estimate lamellar structure in vivo.

The number and thickness of the lamellae have a non-negligible impact on disc mechanics (Adam et al. 2015). Therefore, measuring lamellae in vivo would represent an improvement for the personalization of numerical models.

Ultrasonography has shown potential in the assessment of fibrous tissues. For instance, it has been used to examine healthy and pathological tendons, both to characterize their structural (Denoix et al. 1990; van Schie et al. 1999) and mechanical properties (Crevier-Denoix et al. 2005). Feasibility of ultrasonographic imaging of the disc has been tested preliminary in vitro (Johnson et al. 2002; Kakitsubata et al. 2005) and in vivo with a posteromedial approach (McNally et al. 2000). Recent technical advances allowed a vast improvement in ultrasonographic image quality and resolution, but applications to the intervertebral disc are still lacking. In particular, it was never determined, to our knowledge, whether the concentric structures that are visible in ultrasonographies indeed correspond to the lamellae.

Magnetic resonance imaging (MRI), on the other hand, was previously used to characterize disc lamellar structure in vitro (Wright et al. 2016; Sharabi et al. 2018), while polarized light microscopy remains the gold standard to observe lamellae (Adam et al. 2015). Thus, these two validated modalities could be used to interpret ultrasonographic images in vitro.

The hypothesis of the present work was that current ultrasonographic technology could give access to the characterization of the AF lamellar structure in vivo. The aim was to demonstrate the feasibility of such technique by measuring lamellar thickness in a cohort of healthy adolescents and comparing the results with literature values of in vitro measurements. Moreover, to further validate the information conveyed by the ultrasound images, discs from cow tails were imaged with ultrasound, MRI and optical microscopy to compare the lamellar structure visible in each modality.

## Materials and methods

### Subjects

Healthy adolescents, ageing between 10 and 18 years of age, with no antecedents of musculoskeletal disease were included after signed informed consent (theirs and their parents when minor). Their weight and height were recorded, and their body mass index (BMI) was calculated. The study was approved by the ethical committee (C.P.P Île de France VI 14 409).

### In vivo ultrasonography

**Fig. 1:**
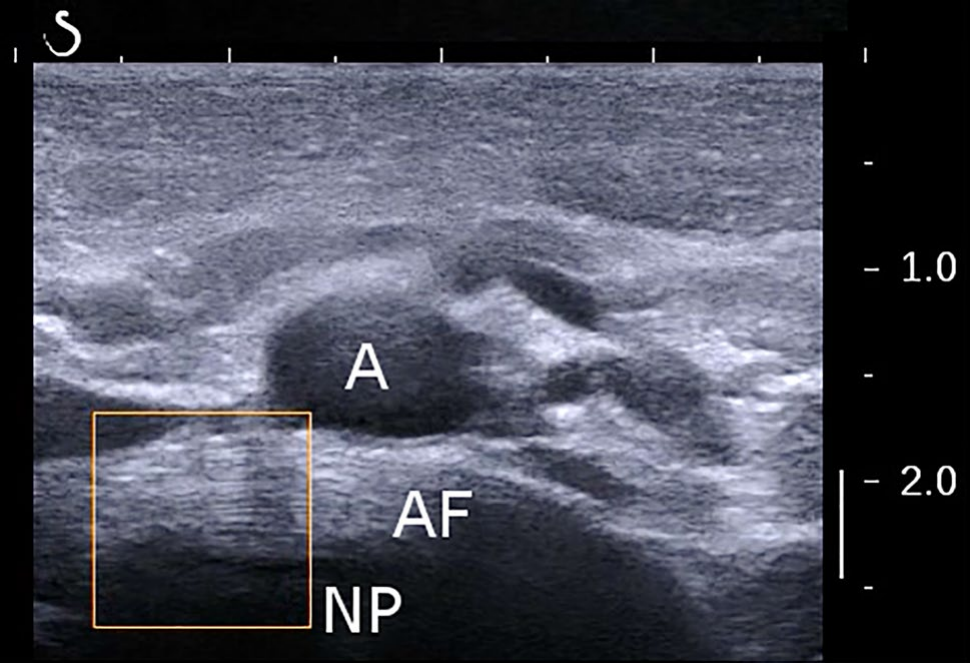
Example of lamellar structure of the annulus fibrosus (AF) of L3–L4 intervertebral disc. *A* aorta, *NP* nucleus pulposus

Ultrasonography was performed by an experienced user with an Aixplorer (SuperSonic Imagine, Aix-en-Provence, France) and a linear SL 10-2 probe. Imaging protocol was previously described for shearwave elastography of the disc (Langlais et al. 2018): the subject was supine, arms along the body, in normal respiration. The probe was placed against the abdomen, oriented in the transversal plane. Disc L3-L4 was detected by looking for the abdominal aortic bifurcation, which is usually at the L4 vertebral level (Deswal et al. 2014). The orientation of the probe was then carefully adapted to obtain a good image of the AF lamellar structure (Fig. 1).

### Measurement of lamellar thickness

**Fig. 2:**
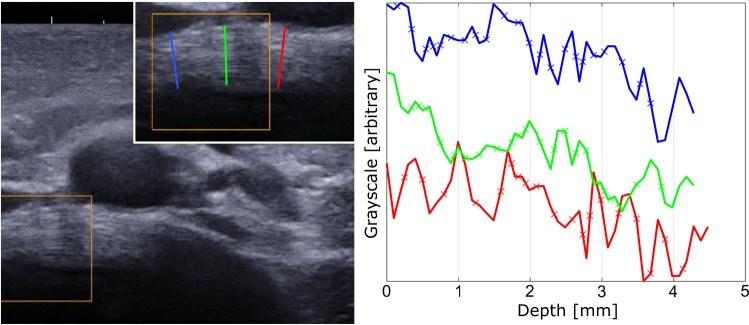
Example of extraction of lamellar profile in a lumbar annulus fibrosus. Three radial lines were defined by an operator across the annulus fibrosus, one medial and two mediolateral. Grayscale values of the pixels along each line (plot) were extracted to automatically detect the interlamellar borders (crosses), which were defined as the horizontal midpoints between main peaks and valleys

Image processing was performed with ImageJ (Schneider et al. 2012). An operator traced three radial lines across the AF, one medial and two mediolateral (Fig. 2), between the annulus outer and inner surface; the outer ending of the line was defined on the disc’s outer border, while the inner ending corresponded with the last visible lamella (Fig. 2). The lines were spaced by about 5 mm. Grayscale values of the pixels along each line were extracted and automatically processed in Matlab 2016b (The MathWorks Inc., Natick, MA) to detect the interlamellar borders. These were defined as the midpoint between each peak and valley. Finally, the average lamellar thickness for each AF was calculated as the average distance between interlamellar distances.

Conversion of pixel size to millimeter was possible thanks to the vertical scale available in each image (Fig. 1).

### In vitro validation

A cow tail was retrieved at the local butcher. The first two caudal discs were imaged with ultrasound applying a previous described protocol (Vergari et al. 2014a), similar to the one adopted for the in vivo measurements: the probe was gently placed on the ventral aspect of the cow tail with abundant acoustic gel, oriented transversally relative to the tail’s main axis. The probe was then tilted to image the disc’s transversal plane and acquire an image of the annulus’ lamellar structure.

Afterwards, the tail was frozen intact to preserve disc microstructure and hydration, and it was thawed at room temperature on the day of the subsequent analyses.

MR images of the same two discs were acquired with a Gyroscan Intera 1.5 T whole-body imager (Philips, Amsterdam, Netherlands) using a 47-mm microscopy coil. The MRI acquisitions were realized in two phases. First, a fast and low-resolution longitudinal scan was performed to precisely determine the transversal midplane of each disc. Then, high-resolution scans (TR = 80 ms, TE = 21 ms, NSA = 20, Resolution = 90 × 90 × 200 μm) were performed to obtain high-resolution MR images of this plane.

On the same day, the cow tail was dissected to expose the first two discs. A wedge was excised from the ventral part of each annulus, and it was placed in a cryo-microtome. Slices of 30 μm thickness were cut, they were placed on a microscope slide and then they were imaged with a Nikon Eclipse E200 microscope, fitted with a 4x/0.10 Nikon objective, two cross-polarizer filters placed before and after the sample and a QImaging Retiga 2000R camera with a definition of 1600 × 1200 pixels. A rectangular grid of images was acquired to cover the whole sample, and they were stitched together with ImageJ’s plugin MosaicJ (Thévenaz and Unser 2006). Each annulus yielded a microscopic image of approximately 4 × 4 mm with a pixel size of 1.86 μm.

The images of each annulus, which were obtained with the three modalities, were resampled to the same pixel size and manually superimposed to appreciate the consistency of the annulus appearance across modalities.

### Reliability and statistics

A second operator processed the in vivo images of a random subset of 10 subjects. Reliability of lamellar thickness measurement was assessed by calculating the root mean squared difference between operators and the intraclass correlation coefficient (ICC).

Data distribution of lamellar thickness was not normal (Lilliefors test, *p* < 0.05), so correlations were analysed with Spearman’s rank test and differences between sexes with Mann–Whitney tests. Statistical significance was set at *p* < 0.05.

## Results

Twenty-nine adolescents were included; Table 1 reports their demographical data. One subject (not included in Table 1) was excluded since his abdomen was too muscular and lean, and reliable acoustic contact could not be achieved.

**Table 1:** Anagraphical data

	Adolescents (*n* = 29)
Age	13 ± 1.9 (range 10–16)
Sex	13 girls, 16 boys
Height (cm)	160 ± 10 (range 140–180)
Weight (kg)	48.2 ± 11.3 (range 30–73)
Body mass index	18.6 ± 3.1 (range 12.6–27.6)

Average lamellar thickness was 229.7 ± 91.5 μm, ranging between 156 and 323 μm. Within-subject lamellar variability was 17.8 μm in terms of twice the standard deviation. In other words, 95% of the measured lamellae in each patient were within the range [average ± 17.8 μm].

Root mean squared difference between operators was 17 μm (7% of overall average), while ICC was 0.7, indicating “good agreement” between operators.

A significant correlation was observed between lamellar thickness and BMI (*p* = 0.0058, rho Spearman = 0.29). However, when BMI was substituted with BMI percentile according to subject age and sex (using World Health Organization Child Growth Standards), this correlation disappeared (*p* = 0.7).

**Fig. 3:**
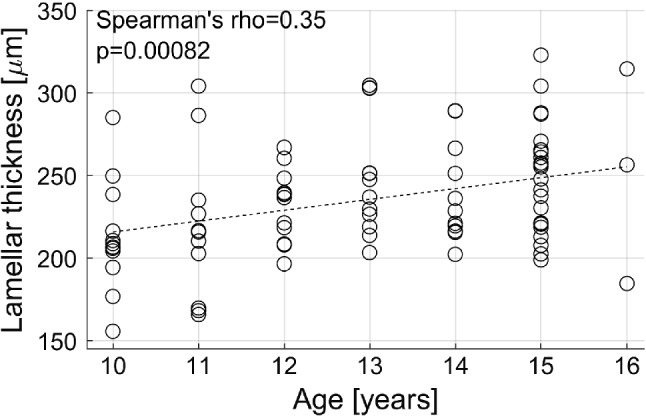
Correlation between age and lamellar thickness

Lamellar thickness was also correlated to subject age (*p* = 0.0082, rho Spearman = 0.35, Fig. 3): it was 220.8 ± 33.9 μm before 13 years and 245.4 ± 34.0 μm after this age (*p* = 0.002). No difference was observed between sexes.

Average pixel size in ultrasonographic images was 92 μm, and average imaging time was 5 min.

**Fig. 4:**
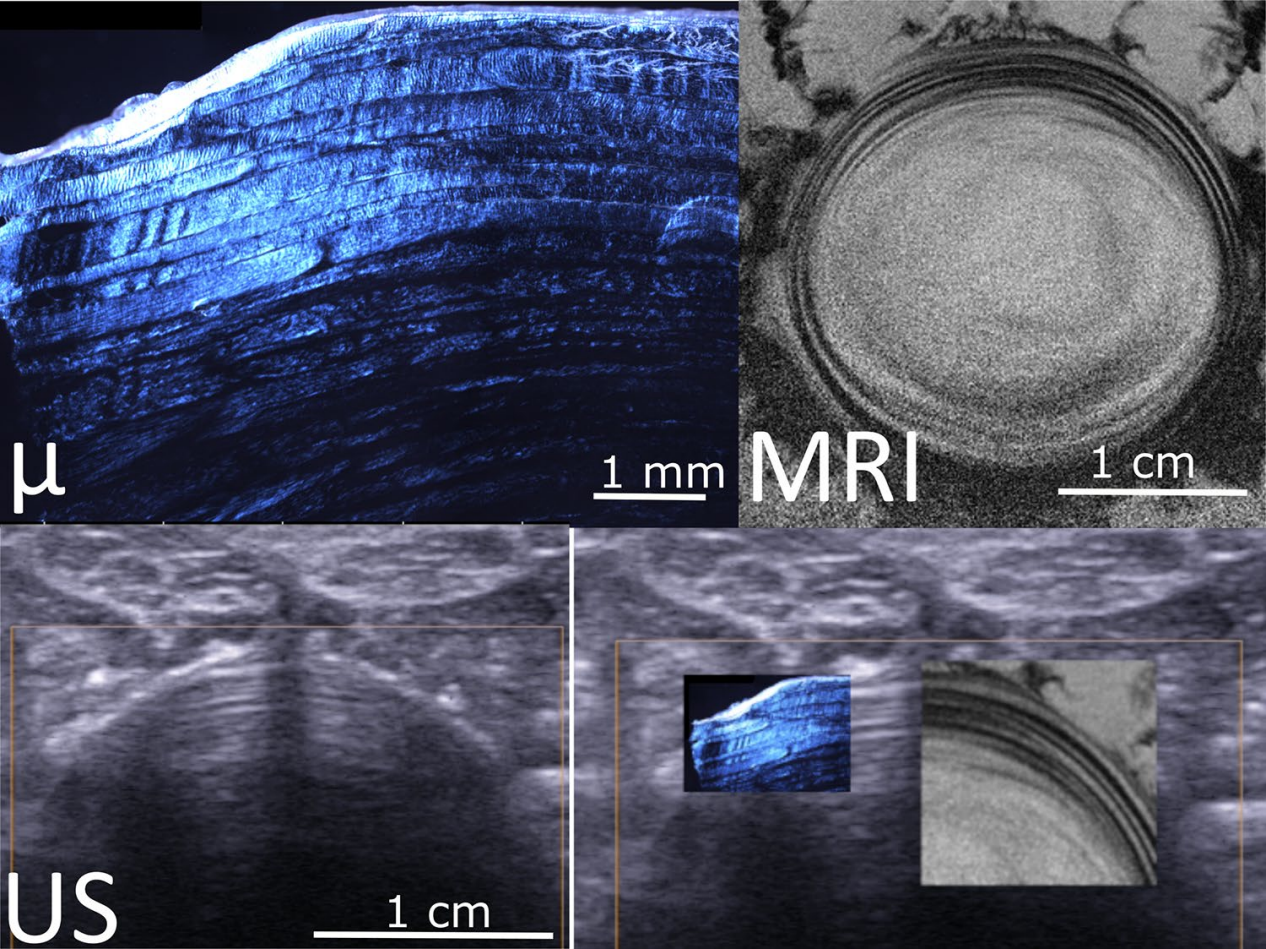
Example of multimodal imaging of annulus structure. Polarized light microscopy (*μ*), magnetic resonance image (MRI) and ultrasonography (US) of a cow tail disc. The fourth panel shows the three images superimposed at the same pixel size; lamellae appear continuous in the three images, confirming that the alternate dark/bright banding visible in US correspond to the lamellae

**Fig. 5:**
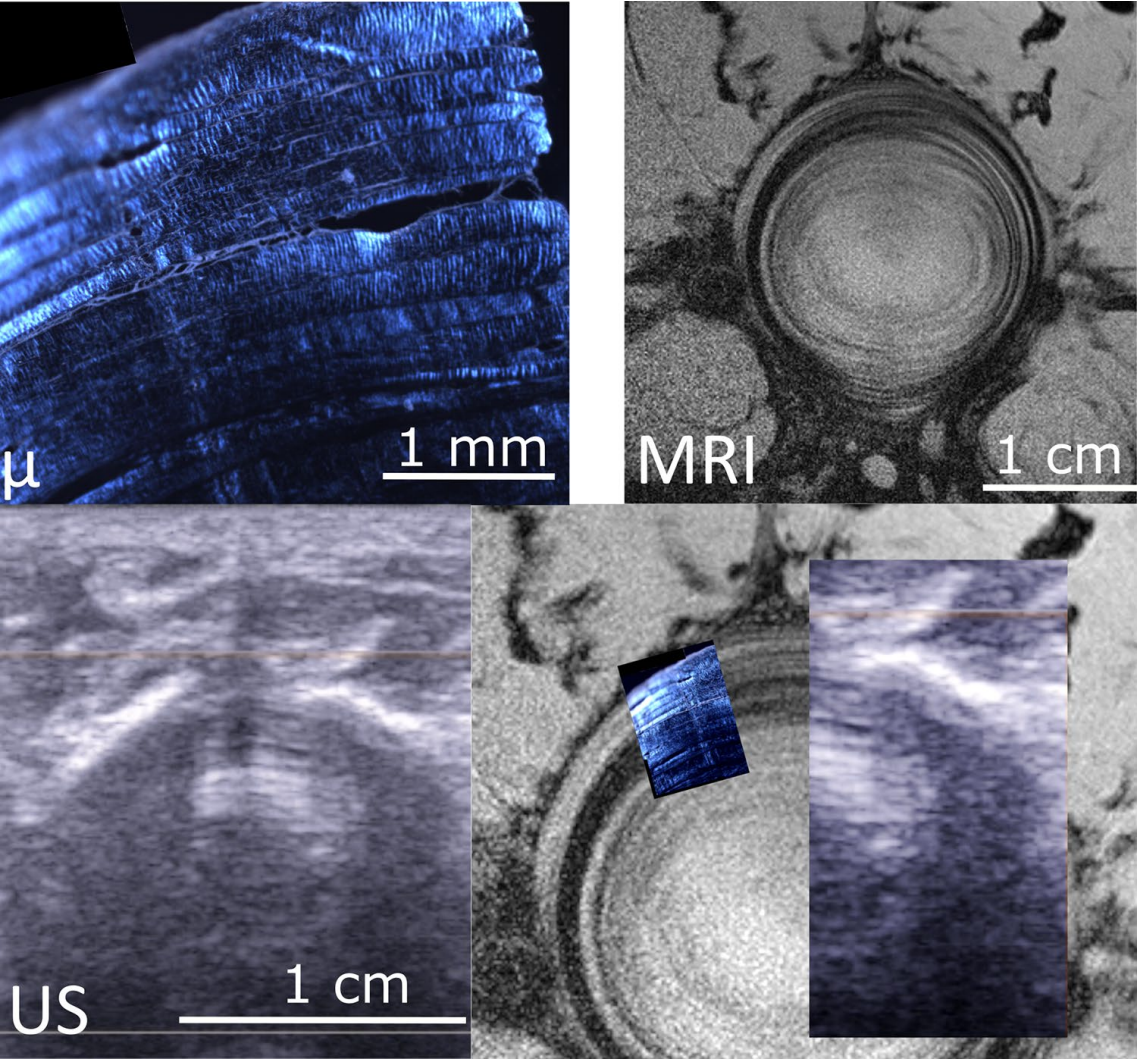
Second example of multimodal imaging of annulus structure. Polarized light microscopy (*μ*), magnetic resonance image (MRI) and ultrasonography (US) of a cow tail disc. The fourth panel shows the three images superimposed at the same pixel size; lamellae appear continuous in the three images, confirming that the alternate dark/bright banding visible in US correspond to the lamellae

Figures 3d and 4d show the superimposition of three imaging modalities for the two first discs from cow tails. The images show good qualitative agreement, with matching lamellar structure (Figs. 4 and 5).

## Discussion

In this preliminary work, the lamellar structure of the intervertebral disc AF was quantified in vivo for the first time, and the information conveyed by ultrasound was validated with multimodality in vitro imaging. The originality of this approach was to test ultrasonography, which is widely used in clinic, to assess the intervertebral disc with a clinic-compatible protocol and a quantitative analysis. Lamella thickness of L3/L4 annulus fibrosus, which was non-invasively measured with ultrasonography, was similar to previously reported values for adult discs measured in vitro: 229.7 ± 91.5 μm in the present study (10–16 years old) against 180 ± 20 μm in young adults (18–29 years old) and 420 ± 60 μm in adults (53–76 years old) (Marchand and Ahmed 1990).

The learning curve for in vivo AF imaging is not long, but care must be taken to obtain good-quality images. The adolescent must be relaxed, and steady pressure must be applied to the probe to move aside intestinal contents and gas. It is advisable to first detect the aortic bifurcation, which corresponds the L4 vertebra in 64% of subjects (Deswal et al. 2014). Then the probe can slide cranially to measure L3-L4 disc, as was done in the present work, or caudally, to measure the lower discs. In some cases, the probe must be placed slightly medially, if the navel produces shadow artefacts in the image, although in general, a large amount of gel is enough to fill the navel and avoid artefacts.

The lamellar structure of the AF is only visible if the probe is in the same plane of the disc; otherwise, ultrasound waves cross a small part of the AF but then they are immediately reflected by the adjacent vertebral bodies. Therefore, once the disc position has been identified, the probe must be slowly titled until the lamellar structure appears. In other words, seeing the lamellar structure in the image means that the probe is in the correct orientation relative to the disc and that the imaging plane is between the adjacent vertebral bodies. This can be further confirmed with the in vitro measurements, where the intervertebral disc can actually be seen during the acquisition, and the probe orientation can be estimated relative to it.

Beyond research applications, such characterization could also have interesting clinical applications since measurement is rapid, non-invasive and potentially accessible in clinical routine. Evidence is accumulating on the relationship between disc alterations and spine deformity, such as adolescent idiopathic scoliosis (AIS), although the pathogenesis and natural progression of this pathology are still poorly understood (Kouwenhoven and Castelein 2008; Yagi et al. 2014). For instance, increased intervertebral torsion is characteristic of the progressive scoliosis geometric phenotype (Skalli et al. 2017), suggesting a role of altered disc torsional behaviour, which in turn can be affected by lamellar structure (Adam et al. 2015). Moreover, it is known that scoliotic spines are stiffer than healthy ones (Deviren et al. 2002), and recent in vivo measurements showed that this increased stiffness could be due to the AF (Langlais et al. 2018). Mechanical and microstructural alterations of the disc have been already demonstrated in adolescent idiopathic scoliosis patients (Yu et al. 2005; Langlais et al. 2018). If these changes are accompanied by morphological ones, the technique tested in this work could be used to assess them. Other structural alterations of the AF, such as delamination or focal lesions, could be the cause or the consequence of a degenerative process, pathologic deformity or trauma (Naish et al. 2003), and they could be detected by ultrasound.

In perspective, non-invasive assessment of disc microstructure could have vast potential clinical applications, since it could represent a novel tool to assess scoliosis or disc degeneration. Accessing disc structure in vivo could also have a huge impact on numerical modelling of the disc (Adam et al. 2015), but also of the whole scoliotic trunk, given the increasing interest in multiscale modelling approaches (Viceconti et al. 2015). Further technical and methodological advances of MRI could make it possible to cross-validate the proposed technique with in vivo measurement of disc lamellar structure. Moreover, peri-operative imaging of the disc during spinal surgery would remove potential artefacts due to the tissues surrounding the spine, thus providing further validation of the technique.

In vitro validation of the technique was limited to two samples and to a qualitative comparison. Still, multimodality imaging of animal samples confirmed that the fibrous concentric structures visible in ultrasound are indeed lamellae (Figs. 4, 5). Polarized light microscopy shows individual lamellae with clearly visible layers of fibres separated by interlamellar matrix. MRI and ultrasonography resolution do not allow seeing fibres within each lamella, but they show lamellae as a pattern alternate bright and dark layers. It was previously demonstrated that signal intensity of each layer in MRI images depends on the orientation of the fibres within the layer, as they interact with the magnetic field (Wright et al. 2016). More fundamental research is needed to determine what causes the lamellae to be bright or dark in ultrasound images, but a similar phenomenon might be occurring when ultrasound waves interact with the lamellae.

This work is a preliminary feasibility study; the main limitation is that segments were manually defined on the AF to estimate lamellar thickness. Such approach suffers from the subjective placement of the segments by of the operator, although the same principles were applied for all images (two mediolateral and one medial segments, between the outer and inner surfaces of the AF). Work is underway to develop a semi-automatic method to extract lamellar thicknesses and distribution. The second limitation is that in vivo measurement was only performed in the lumbar region of adolescents. Thoracic discs are hidden anteriorly by the rib cage and posteriorly by the intervertebral joints and neural arch. Ultrasound-based elastographic measurement of adult cervical disc was previously demonstrated feasible (Vergari et al. 2014b); however, it is not yet clear if the quality of b-mode images would be sufficient to characterize lamellar structure.

A correlation was observed between lamellar thickness and subject BMI. However, BMI increases with age, and its interpretation depends on subject sex. Therefore, comparing lamellar thickness with the percentile-equivalent of BMI according to subject characteristics seemed more appropriate; when this correction was applied, the correlation disappeared. Therefore, the relationship between lamellar thickness and BMI could be indirect, and actually depending on subject age.

Marchand and Ahmed reported an effect of age on the thickness and on the number of distinct layers found in the annulus: older subjects showed less layers, and the layers were thicker. This corroborates the results obtained in the present work where a positive correlation was shown between age and lamellar thickness (Fig. 3). Interestingly, the age of subjects included in Marchand and Ahmed’s study and the present one do not overlap: the older patient in the present work was 16 years old, while the younger one in the previous study was 18. This could mean that lamellae tend to thicken throughout the subject’s life, the later phase of which could be associated with a loss of collagen organisation which has been described in older adults (Gruber and Hanley 2002).

While this study aimed at determining the feasibility of ultrasound characterization of AF lamellar structure, further work could give an insight on the development and progression of disc alteration. The non-invasive character of ultrasonography opens the way to large-scale data collection which could clarify the role of the disc in AIS.
